# Role of UFMylation in tumorigenesis and cancer immunotherapy

**DOI:** 10.3389/fimmu.2024.1454823

**Published:** 2024-08-23

**Authors:** Li-juan Ding, Xin Jiang, Te Li, Shudong Wang

**Affiliations:** ^1^ Department of Radiation Oncology, The First Hospital of Jilin University, Changchun, Jilin, China; ^2^ Department of Geriatrics, The First Hospital of Jilin University, Changchun, Jilin, China; ^3^ Department of Cardiology, The First Hospital of Jilin University, Changchun, Jilin, China

**Keywords:** Ufmylation, oncogenesis, treatment, immunotherapy, cancer

## Abstract

Protein post-translational modifications (PTMs) represent a crucial aspect of cellular regulation, occurring after protein synthesis from mRNA. These modifications, which include phosphorylation, ubiquitination, acetylation, methylation, glycosylation, Sumoylation, and palmitoylation, play pivotal roles in modulating protein function. PTMs influence protein localization, stability, and interactions, thereby orchestrating a variety of cellular processes in response to internal and external stimuli. Dysregulation of PTMs is linked to a spectrum of diseases, such as cancer, inflammatory diseases, and neurodegenerative disorders. UFMylation, a type of PTMs, has recently gained prominence for its regulatory role in numerous cellular processes, including protein stability, response to cellular stress, and key signaling pathways influencing cellular functions. This review highlights the crucial function of UFMylation in the development and progression of tumors, underscoring its potential as a therapeutic target. Moreover, we discuss the pivotal role of UFMylation in tumorigenesis and malignant progression, and explore its impact on cancer immunotherapy. The article aims to provide a comprehensive overview of biological functions of UFMylation and propose how targeting UFMylation could enhance the effectiveness of cancer immunotherapy strategies.

## Introduction

Protein post-translation modification (PTM), a kind of chemical modification of a protein, occurs after proteins are translated from messenger RNA (mRNA) (1, 2). PTMs play an important role in regulation of protein function via controlling its localization, stability and interactions with other proteins, leading to regulation of various cellular processes upon different internal and external signals (3). Dysregulation of PTMs leads to various diseases, including cancer, inflammatory diseases and neurodegenerative disorders (4). Better understanding the mechanisms of PTMs could help us identify potential therapeutic targets for diverse diseases. There are more than 400 types of PTMs, which affect protein functions (5). The common types of PTMs include phosphorylation (addition of a phosphate group) (6), ubiquitination (addition of ubiquitin molecules) (7, 8), acetylation (addition of an acetyl group) (9), methylation (addition of a methyl group) (10), glycosylation (addition of carbohydrate group) (11), Sumoylation (addition of small ubiquitin-like modifier) (12, 13), palmitoylation (addition of palmitic acid) (14).

In recent years, UFMylation has been emerged to regulate various cellular processes, such as protein stability, cellular stress responses to different internal and external signals, and cellular signaling pathways that regulate cellular biological functions (15, 16). UFMylation belongs to a PTM and involves the covalent attachment of ubiquitin-fold modifier 1 (UFM1) to target proteins (17). UFMylation typically involves three important enzymatic steps, activation, conjugation and ligation (18, 19). UFM1 is activated by an E1 activating enzyme to initiate UFMylation, which prepares it for transfer to the next enzyme. Then, the activated UFM1 is transferred to an E2 conjugating enzyme, which acts as an intermediary carrier in the UFMylation process. Lastly, an E3 ligase enzyme promoted the transfer of UFM1 from the E2 enzyme to the target protein, which completes the UFMylation process and influencing the function of the target proteins (20). There are similarities between UFMylation and ubiquitination. Both UFMylation and ubiquitination are PTMs where small proteins (UFM1 and ubiquitin) are covalently attached to target protein. Both processes involve a series of enzymatic steps by E1, E2 and E3 enzymes. Both UFMylation and ubiquitination are reversible processes because specific proteases can remove UFM1 or ubiquitin from target proteins. Moreover, both modifications can alter the function, localization, interaction and stability of proteins. Evidence has shown that UFMylation is involved in numerous cellular functions, such as DNA damage response, endoplasmic reticulum (ER) stress response, and the regulation of the immune system (21). Hence, in this review, we discuss the role of UFMylation in tumorigenesis and progression. Moreover, we describe the functions of UFMylation in cancer immunotherapy. Targeting UFMylation could be useful for improving cancer immunotherapy.

## UFM1 conjugation system

**Figure 1:**
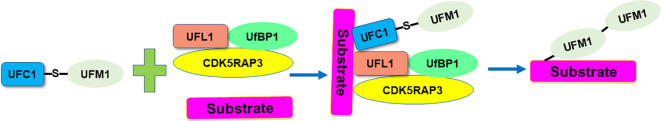
Illustration of the UFMylation process.

UFM1 is a novel ubiquitin-like molecule (UBL) with evolutionary presence across multiple species. Like ubiquitin, UFM1 undergoes a sequential three-step enzymatic process for covalent attachment to its substrates. This process involves the UFM1-activating enzyme (E1), which is known as UBA5 (ubiquitin-like modifier-activating enzyme 5), the UFM1-conjugating enzyme 1 (E2, UFC1), and the UFM1-specific ligase 1 (E3, UFL1) (21). Specifically, UBA5 is a key enzyme involved in the UFMylation pathway, which functions similarly to an E1 enzyme in the ubiquitin system, catalyzing the initial step of activating the ubiquitin-like protein UFM1. UBA5 first activates UFM1 by forming a high-energy thioester bond between the C-terminal glycine of UFM1 and the cysteine residue in the active site of UBA5. This step is ATP-dependent, requiring energy to activate UFM1 for subsequent conjugation. After activation, UFM1 is transferred from UBA5 to the UFC1, which is crucial for the next step in attaching UFM1 to target proteins (22). UFL1 acts as a ligase, specifically an E3 ligase in the UFMylation process, to catalyze the final step of attaching UFM1 to target proteins. De-UFMylation is carried out by specific enzymes UFSPs (UFM1 specific peptidases). The proteases UFSP1 and UFSP2 involve in the maturation of the UFM1 precursor and de‐UFMylation process. These enzymes reverse the attachment of UFM1 to target proteins, thereby regulating the functions and interactions of these proteins. DDRGK1 (DDRGK domain containing 1) and CDK5RAP3 involve in regulation of the UFMylation system. UFBP1 (UFM1-specific binding protein 1) interacts with UFM1 and the E3 ligase UFL1, helping to facilitate the conjugation of UFM1 to substrate proteins (**Figure 1**).

RCAD/UFL1 plays roles in various cellular processes, including ER signaling, the UPR, and neurodegeneration (22, 23). One study reveals that RCAD/UFL1 is critical for embryonic development, HSC survival, and erythroid differentiation. Deletion of RCAD/UFL1 significantly impairs hematopoietic development, leading to severe anemia, cytopenia, and eventual lethality. Depletion of RCAD/UFL1 induces elevated endoplasmic reticulum stress and activates the UPR in bone marrow cells. Additionally, the absence of RCAD/UFL1 disrupts autophagic degradation, increases mitochondrial mass and ROS (reactive oxygen species), and triggers a DNA damage response, p53 activation, and heightened cell death in HSCs (hematopoietic stem cell). This study supports the essential role of RCAD/UFL1 in murine hematopoiesis and development via maintaining cellular homeostasis (24). The UFMylation pathway that is facilitated by UBA5 influences various cellular processes including protein stability, cellular stress responses, and cellular signaling pathways. Dysregulation of UFMylation pathway can contribute to disease mechanisms, particularly in neurodegenerative diseases and cancer. In the following paragraphs, we will describe the mechanisms by which aberrant UFMylation contributes to tumorigenesis in various cancer types.

## UFMylation in various cancer types

Liu et al. reported UFMylation kept p53 stability via antagonizing its ubiquitination (25). It is clear that p53, which is known as the “guardian of the genome”, is a crucial protein known primarily for its important role in preventing cancer (26, 27). Tumor suppressor p53 has been reported to regulate cell cycle, DNA repair, apoptosis and response to stress (28). Mutations of p53 have been observed in many cancer types and involves in tumorigenesis, suggesting that understanding how p53 works and how its activity is controlled or altered in cancer cells is critical for developing new therapeutic strategies (29). Evidence has suggested that p53 can be regulated by PTMs (30). Liu and coworkers found that UFM1 can covalently modify p53, which stabilizes the protein by reducing its ubiquitination and subsequent proteasomal degradation. Mechanistically, UFL1 competes with MDM2 (mouse double minute 2 homolog) for binding to p53, thereby stabilizing it. Knockdown of UFL1 or DDRGK1 leads to decreased p53 stability, enhanced cell growth, and tumor formation *in vivo* (25). Moreover, both UFL1 and DDRGK1 are downregulated in a significant proportion of renal cell carcinomas, where their expression levels positively associated with p53 levels. These findings underscore UFMylation as a vital PTM that maintains p53 stability and tumor-suppressive activity, suggesting that targeting UFMylation could be a viable therapeutic strategy in cancer treatment. In the following sections, we will describe the various role of UFMylation in different cancer types (25).

### Breast cancer

Breast cancer is one of the most common cancers among women worldwide (31). The most common forms of breast cancer are ductal carcinoma *in situ* and invasive ductal carcinoma (32). Triple-negative breast cancer (TNBC) is a subtype of breast cancer characterized by the absence of three common receptors: estrogen receptors (ER), progesterone receptors (PR), and human epidermal growth factor receptor 2 (HER2) (33, 34). TNBC accounts for about 10-20% of all breast cancers. TNBC does not respond to hormonal therapy (such as tamoxifen or aromatase inhibitors) or therapies that target HER2 receptors, such as Herceptin (trastuzumab) (35, 36). Hence, TNBC tends to be more aggressive and has a higher probability of metastasis, particularly in the first few years after diagnosis. Factors that can increase the risk of breast cancer include age, genetic mutations (such as BRCA1 and BRCA2), family history of breast cancer, radiation exposure, obesity, etc. (37). Common treatments for breast cancer include surgery, radiation therapy, chemotherapy, hormone therapy, targeted therapy and immunotherapy (38–40). Understanding breast tumorigenesis is essential for prevention, early detection, and effective treatment strategies, which significantly improve the prognosis (41).

Yoo et al. discovered that the UFMylation of the nuclear receptor coactivator ASC1 (activating signal cointegrator 1) is critical for the activation of estrogen receptor alpha (ERα) in response to 17β-estradiol (E2). In the absence of E2, ASC1 remains non-modified due to its association with the UFM1-specific protease UfSP2. Upon E2 exposure, ERα displaces UfSP2 from ASC1, leading to its UFMylation. This modification enhances the interactions between ASC1 and other coactivators such as p300 and SRC1 at ERα-responsive gene promoters (42). Manipulating ASC1 levels or its UFMylation status profoundly influences ERα-driven tumor progression. Specifically, ASC1 overexpression or reduced UfSP2 expression promotes tumor development, an effect mitigated by tamoxifen. Conversely, expression of a UFMylation-deficient ASC1 variant or suppression of the UFM1-activating enzyme UBA5 inhibits tumor growth. These findings highlight the pivotal role of ASC1 UFMylation in modulating breast cancer development via ERα transactivation, presenting novel insights into the regulatory mechanisms of hormone-responsive breast cancer (42).

Yoo et al. further found that ERα is modified by UFM1, and this UFMylation substantially enhances ERα stability and transactivation capabilities. Specifically, suppressing UFSP2 significantly increases ERα stability by reducing its ubiquitination. Conversely, ERα stability is compromised by inhibiting UFMylation through the silencing of UBA5 (43). Furthermore, this group identified Lys171 and Lys180 on ERα as primary UFM1 acceptor sites, and show that substituting these lysine residues with arginine (2KR mutation) drastically diminishes ERα stability. Furthermore, this 2KR mutation impedes 17β-estradiol-induced transactivity of ERα and downregulates the expression of key downstream targets such as pS2, cyclin D1, and c-Myc, indicating the necessity of UFMylation for transcriptional function of ERα. The 2KR mutation also inhibits anchorage-independent colony growth in MCF7 cells. Importantly, components of the UFM1 conjugation machinery, including UBA5, UFC1, UFL1, and UFBP1, are markedly elevated in ERα-positive breast cancer cell lines and tissues. These results underscore the vital function of ERα UFMylation in enhancing its stability and transcriptional activity, thereby promoting breast cancer progression (43).

### Glioblastoma

Glioblastoma, which is often called glioblastoma multiforme (GBM), is one of the most aggressive types of primary brain cancer in adults (44). Glioblastoma is highly malignant due to its rapid growth and tendency to invade surrounding brain tissue. Glioblastomas have high level of cellular diversity because they contain a mix of cell types, making them difficult to treat (45). Symptoms of glioblastoma can vary depending on the location of the tumor in the brain but typically include headaches, nausea, seizures, cognitive impairments, and neurological deficits such as weakness or sensory changes (46). MRI (magnetic resonance imaging) and CT (computed tomography) scans are used to diagnose glioblastomas, which can detect the size and location of the tumor. A biopsy is used to confirm glioblastoma diagnosis (47). Clinically, treatment for glioblastoma involves a combination of surgery, radiation therapy, immunotherapy and chemotherapy (48–50). In general, after surgery removes tumor as much as possible, radiation therapy in combination with chemotherapy kill any remaining cancer cells (51). Prognosis of patients with glioblastoma remains poor with median survival times ranging from 12 to 18 months. Understanding the genetic and molecular mechanism underlying glioblastoma is critical to develop more effective treatments that target specific and important pathways in glioblastoma oncogenesis and resistance to current chemotherapy, such as temozolomide (52, 53).

One study used genome-wide CRISPR-Cas9 approach explored genetic vulnerabilities in glioblastoma stem cells (GSCs). Moreover, this group explored the mechanisms of temozolomide sensitivity in GSCs (54). Utilizing CRISPR-Cas9 in patient-derived GSCs, the entire coding genome was investigated to identify critical pathways that drive growth and underpin the gene-essential circuitry of GBM stemness and cell proliferation. This group highlighted the significance of SOCS3 (suppressor of cytokine signaling 3), USP8 (ubiquitin specific peptidase 8), DOT1L (disruptor of telomeric silencing 1-like), which belong to the SOX transcription factor family. Moreover, protein ufmylation is critical in promoting GSC growth. Specifically, all members of the ufmylation pathway, including UBA5, UFC1, UFL1, and UFSP2, were crucial, as single-gene knockouts significantly reduced cell fitness. This highlights each step in this pathway as a potential therapeutic target (54). Treatment of 14 patient-derived GSC cultures with DKM 2-93, which is an inhibitor of UBA5, demonstrated strong anti-proliferative effects. Additionally, depletion of UFC1 and UFSP2 promoted CHOP expression, an ER-stress response marker, suggesting a connection to ER homeostasis maintenance. The identification of SEL1L and HRD1 as GBM-specific fitness genes further underscores the potential of targeting proteostasis networks in therapeutic strategies for GBM. These findings point to the UFMylation pathway as a critical component for GBM stem cell maintenance and a promising target for developing new GBM treatments. Furthermore, this study uncovered mechanisms of temozolomide resistance, suggesting potential avenues for combination therapy. This genome-wide functional approach enhanced the understanding of GBM growth dynamics and drug resistance (54).

### Renal cancer

Renal cancer, which is often known as kidney cancer, is one of the common cancers in human. The most common type of renal cancer is renal cell carcinoma (RCC), which makes up about 90% of all kidney cancers (55). Within RCC, there are several subtypes, including clear cell, papillary, and chromophobe renal cell carcinomas. Several risk factors for renal cancer development have been reported, including smoking, obesity, high blood pressure, family history of renal cancer, and genetic conditions (56). Diagnostic approaches of renal cancer include ultrasound, CT scans, MRI and sometimes biopsy (57, 58). Treatments for renal cancer include surgery, cryoablation, radiation therapy, targeted therapy and immunotherapy (59). Patients with localized renal cancers often have a high cure rate with surgery, while patients with advanced and metastatic renal cancers often have a poor prognosis (60). Advancements in the understanding of molecular biology of renal cancer could develop novel treatments and extend patient survival. Wang et al. reported that UFMylation is activated in renal cancer (61). Our group investigated the relationship between UFMylation, autophagy, and the UPS in renal cancer. UFMylation levels remained unchanged with either activation or inhibition of autophagy, as evidenced by consistent LC3 conversion rates in renal cancer tissues compared to adjacent non-cancerous tissues. This suggests that the increase in UFMylation observed in renal cancer is independent of autophagy. Inhibition of the UPS with MG132 led to increased UFMylation in renal cancer cells, indicating a potential association between UFMylation and UPS activity. Additionally, UFMylation levels did not correlate with VHL gene mutations, which is frequently mutated in renal cancer and serves as a key E3 ligase in the UPS (62). Moreover, treatment with sunitinib, targeting multiple tyrosine kinases, significantly reduced UFMylation levels, while upregulation of active UFM1 was found to partially counteract the anti-tumor effects of sunitinib. These findings suggest that UFMylation might serve as a novel molecular target for the treatment of renal cancer (61).

### Colon cancer

Colon cancer is one of the most common types of cancer in both men and women. Numerous factors that can increase the risk of colon cancer include older age, family history of colon cancer or polyps, inflammatory intestinal conditions, such as ulcerative colitis or Crohn’s disease, a diet low in fiber and high in fat and calories, diabetes, obesity, smoking, and alcohol (63, 64). Colonoscopy and biopsy are helpful for colon cancer diagnosis. Treatments for colon cancer have surgery, radiation therapy, chemotherapy and targeted therapy (65). Colon cancer can be highly treatable if it is caught early via awareness, colonoscopy screen (66). Zhou et al. performed genomic profiling of the UFMylation family genes and discovered a tumor suppressor UFSP2 in colon cancer (67). This study utilized TCGA data cohort and analyzed the genomic alterations of eight UFMylation family genes, including UFSP1, UFSP2, UFM1, UFL1, UFC1, DDRGK1, UBA5 and CDK5RAP3. Moreover, 55 recurrent and focal SCNA events were uncovered in UFMylation family genes in 33 cancer types. UFSP2 gene was often deleted in 14 cancer types. The frequency for copy number loss of UFSP2, UFM1 and UFL1 was 31%, 31% and 18%, respectively. The frequency for copy number gain for UFC1, UFSP1 and DDRGK1 was 34%, 34% and 30%. Notably, UFSP2 copy number was heterozygous loss in cancer tissues and tumor cells, suggesting a possible haploinsufficiency of the UFSP2 gene. 11% of TCGA samples displayed high-level copy number alterations in at least one UFMylation gene (67). These alterations typically manifested in a mutually exclusive pattern of amplifications and deletions, suggesting that UFMylation genes may have overlapping functions. UFMylation genes generally displayed less than 5% somatic mutations and less than 0.1% transcript fusions. Additionally, RNA sequencing data analysis from the TCGA samples revealed ubiquitous expression of UFMylation genes across various cancer types. Moreover, UFSP2 mutation is frequently observed in colon cancer and uterine corpus endometrial carcinoma. This pattern, along with high recurrent copy number loss, indicate that UFSP2 may perform tumor suppressive function (67). Previous research supports this assertion, showing that knockdown of UFSP2 enhances breast cancer cell growth and tumor formation. Furthermore, significantly reduced levels of UFSP2 mRNA and protein levels were observed in multiple cancer tissues. Knockdown of UFSP2 expression was observed to significantly enhance the growth rates of colon cancer cells *in vitro* and *in vivo*. Additionally, an increase in total UFMylation levels was detected after UFSP2 depletion in cells and xenograft tumors, linking UFSP2 genomic alterations to the functional role of UFMylation in colon cancer (67). GSEA analysis showed that the loss of UFSP2 predominantly affected pathways related to DNA replication, the cell cycle, the spliceosome, the ribosome, and mismatch repair. By knocking down UFSP2 in colon cancer cell lines, there was an increase in the expression of key marker genes, including PCNA and MCM2 for DNA replication, CDK4 and CCND1 for cell cycle regulation, RPL26 for ribosome protein synthesis. These results further substantiate the tumor suppressive properties of UFSP2 in colon cancer (67).

### Pancreatic cancer

Pancreatic cancer has a worse prognosis in human cancer types. This is due to pancreas deep location in the body. In addition, pancreatic cancer often has no symptoms until it has reached an advanced stage, making it difficult to detect early (68). Risk factors for pancreatic cancer have smoking, chronic pancreatitis, diabetes, family history of pancreatic cancer, obesity, etc (69). Treatment options for pancreatic cancer have surgery, radiation therapy, chemotherapy, and targeted therapy (70–72). Because pancreatic cancer has a lower 5-year survival rate, it is important to discover early detection and innovative treatment approaches (73). To achieve this goal, exploration of molecular mechanisms of pancreatic oncogenesis is necessary. One group identified that UBA5 could be a target for pancreatic cancer via chemoproteomic screening of covalent ligands (74). Another group identified that the UFMylation of RPL10 (ribosomal protein L10) contributed to pancreatic adenocarcinoma development (75). The ufmylation of RPL10 was found in both pancreatic cancer cells and tissues. RPL10 UFMylation enhances cell proliferation and promoted cancer cell stemness, primarily through upregulated expression of KLF4 (kruppel-like transcription factor 4). Additionally, mutagenesis of RPL10 UFMylation sites further solidified the link between RPL10 UFMylation and these cellular behaviors, including proliferation and stemness. Overall, UFMylation of RPL10 is a crucial process that promotes the stemness of pancreatic cancer cells, contributing to the progression of pancreatic cancer (75).

### Oral squamous cell carcinoma

Oral squamous cell carcinoma (OSCC) is the most common type of oral cancer. Several risk factors have contributed to OSCC, including tobacco use, heavy alcohol consumption, HPV (human papillomavirus) exposure, chronic dental irritation and dietary factors (76, 77). A biopsy is important for OSCC diagnosis after a clinical examination (78). Imaging test can be used to determine the extent of cancer and tumor invasion, such as X-rays, CT scans, MRI and PET scans (79). Treatments of OSCC have surgery, radiation therapy, chemotherapy or a combination, which are dependent on the tumor stage and location (80, 81). Recently, UFM1 was reported to enhance OSCC progression via regulating immune infiltration (82). Higher expression of UFM1 was found in OSCC and its overexpression was linked to shorter overall survival, indicating that UFM1 could serve as an adverse prognostic factor in OSCC. Furthermore, UFM1 expression could predict poor outcomes in OSCC patients. Functionally, reducing UFM1 expression suppressed the cell proliferation, attenuated cell migration, and invasion in OSCC. Additionally, UFM1 was linked to various immune cells, including Th17 cells, T helper cells, and cytotoxic cells, as well as with processes of ubiquitination. These findings highlight the potential of UFM1 as a biomarker for OSCC prognosis and a target for therapeutic intervention (82).

### Hepatocellular carcinoma

Hepatocellular carcinoma (HCC) is the most common type of primary liver cancer. HCC is often associated with chronic liver disease and cirrhosis. Risk factors of HCC development have chronic hepatitis B or C infection, cirrhosis, alcohol consumption, aflatoxin exposure, nonalcoholic fatty liver disease (NAFLD), etc (83). Besides imaging tests such as ultrasound, CT scans and MRIs, and a biopsy, alpha-fetoprotein (AFP) test in blood may aid in diagnosis (84). Treatments for HCC include surgical resection, liver transplantation, chemoembolization, radiofrequency ablation, targeted therapy drugs and immunotherapy (85–87). Chen et al. reported that lncRNA B3GALT5-AS1 regulated the miR-934/UFM1 axis and inhibited tumor progression in HCC (88). A reduction in B3GALT5-AS1 levels was seen in both HCC cell lines and tissues. Upregulation of B3GALT5-AS1 inhibited the malignant properties of HCC cells. The effects of B3GALT5-AS1 overexpression can be reversed by miR-934 mimics, establishing miR-934 as a downstream effector. Additionally, the impact of miR-934 inhibition was mitigated by UFM1 downregulation, highlighting a link between miR-934 and UFM1. B3GALT5-AS1 suppresses the PI3K(phosphoinositide 3-kinases)/AKT signaling pathway via UFM1. Collectively, B3GALT5-AS1 acts as a potent suppressor of HCC by modulating miR-934 and UFM1, pointing to its potential as a HCC therapeutic target (88). Moreover, loss of UFL1/HFBP1 in hepatocytes activated mTOR pathway and facilitated liver damage and liver carcinogenesis via using hepatocyte-specific Ufl1Δ/Δhep and Ufbp1Δ/Δhep mice (89). High-fat diet (HFD) was used to induce fatty liver disease, and diethylnitrosamine (DEN) was used to trigger liver cancer. Results demonstrated that deletion of Ufl1 or Ufbp1 in hepatocytes led to early signs of liver damage such as hepatocyte apoptosis and mild steatosis by 2 months, progressing to severe hepatocellular ballooning, extensive fibrosis, and steatohepatitis by 6-8 months. Notably, over 50% of the knockout mice developed spontaneous HCC by 14 months. These mice also showed increased susceptibility to HFD-induced fatty liver and DEN-induced HCC. The Ufl1/Ufbp1 complex was found to bind with and inhibit the mTOR/GβL complex, thus reducing mTORC1 activity. Loss of Ufl1 or Ufbp1 dissociated this complex, leading to enhanced mTOR pathway and HCC development. Ufl1 and Ufbp1 play critical roles in preventing liver fibrosis, steatohepatitis, and HCC by acting as inhibitors of the mTOR pathway, highlighting their potential as therapeutic targets for liver diseases (89).

### Gastric cancer

Gastric cancer is one of the common cancer types in the world, which can spread throughout the stomach and to other organs, particularly the esophagus, lungs, lymph nodes, and liver (90). The most common type of gastric cancer is adenocarcinoma, which accounts for about 90% of all gastric cancer cases. Several factors contribute to gastric cancer development, such as helicobacter pylori infection, diet, smoking, family history, chronic gastritis, stomach polyps (91). Gastric cancer is often asymptomatic in the early stages, which can delay diagnosis. Diagnosis typically involves endoscopy, a biopsy and CT scans (92, 93). Treatments for gastric cancer include surgery, chemotherapy, radiation therapy, targeted therapy and immunotherapy (94). The association between the UFM1 expression and the outcomes of gastric cancer patients who had surgery was determined by Lin and coworkers. Lin et al. utilized public databases to explore the relationship between UFM1 and treatment outcomes by single-sample gene set enrichment analysis (ssGSEA). Moreover, the expression of UFM1 was determined in cancerous and paracancerous gastric cancer tissues. UFM1 expression was decreased and interacted with CDK5RAP3. Moreover, CDK5RAP3 and UFM1 expression was inversely associated with one key oncogenic pathway activation, AKT. Low expression of UFM1 and CDK5RAP3 was associated with poor prognosis, which was independent predictor to predict overall survival of gastric cancer patients. Moreover, in combination of UFM1, CDK5RAP3 and TNM (tumor, node, metastasis) stages increased the accuracy of prognosis prediction in gastric cancer patients (95).

Lin et al. further found that UFM1 inhibited the expression of PDK1 via negative regulation of PI3K/AKT signaling, leading to suppression of invasive ability in gastric cancer (96). UFM1 expression was decreased at both protein level and mRNA level in gastric cancer tissues, which was associated with low 5-year survival rate in patients with gastric cancer. Upregulation of UFM1 inhibited migration and invasion abilities in gastric cancer cells, while downregulation of UFM1 exhibited the opposite functions. *In vivo* study further identified the tumor suppressive function of UFM1 in nude mouse model. Mechanistically, UFM1 enhanced the PDK1 ubiquitination level, leading to reduction of the PDK1 protein level, thereby reducing the AKT phosphorylation at Ser473 (96). Another study reported that upregulation of UFBP1 expression correlates with increased progression-free survival in gastric cancer after platinum-based chemotherapy. UFBP1 enhances the sensitivity of gastric cancer cells to cisplatin, whereas its knockdown reduces this sensitivity. Proteomic analysis indicated a significant reduction in aldo-keto reductase 1Cs (AKR1Cs) protein levels due to UFBP1 overexpression. Additionally, UFBP1 modulates ROS production in response to cisplatin. Mechanistic insights showed that UFBP1 decreases AKR1Cs expression and Nrf2 transcriptional activity, promoting Nrf2 degradation via K48-linked polyubiquitination. Further cell and mouse experiments confirmed that UFBP1 amplifies cisplatin sensitivity through the Nrf2/AKR1C axis. These findings identify UFBP1 as a potential prognostic biomarker for gastric cancer and provide a mechanistic basis for personalized chemotherapy approaches (97).

## UFMylation in immunotherapy

Cancer immunotherapy is a type of cancer treatment that harnesses the immune system to fight cancer (98). Although immune cells can recognize and destroy cancer cells, cancer often finds ways to evade immune detection. Immunotherapy aims to boost the immune ability to combat cancer more effectively (99). There are several types of immunotherapy, including checkpoint inhibitors, CAR (chimeric antigen receptors) T-cell therapy, cancer vaccines, monoclonal antibodies (100). Common checkpoint proteins in immunotherapy include PD-1 (programmed death-1), PD-L1 (programmed death ligand-1) and CTLA-4 (cytotoxic T-lymphocyte associated protein 4), which block the immune cells to attack cancer cells (101, 102). CAR T-cell therapy involves modifying T-cells via engineering to produce special receptors on their surface, leading to the better destroying cancer cells (103). Cancer vaccine induces the immune system to target tumor cells, which can help prevent the cancer from growing or recurrence (104, 105). Immunotherapy can be a powerful treatment option for some types of cancer. However, it fails to work for everyone, and figuring out why and how to improve immunotherapy is a major area of research in the field of oncology. Recently, UFMylation has been reported to involve in immunotherapy in cancer (106, 107). Therefore, we describe the role of UFMylation in regulation of immunotherapy in the following paragraphs.

### UFMylation of pirin

Pancreatic ductal adenocarcinoma (PDAC) is the most common type of pancreatic cancer, accounting for about 90% of all pancreatic cancers. PDAC is particularly aggressive with late detection and poor prognosis (37). Treatment options for PDAC depend on the disease stage at diagnosis and may include surgery, chemotherapy, radiation therapy, immunotherapy, or a combination of these approaches (72, 108). One study identified a potent approach to induce ferroptosis in PDAC and stimulate antitumor immune response. Utilizing patient-derived organoid models and a KPC mouse model, which is known as LSL-KrasG12D/+, LSL-Trp53R172H/+, Pdx-1-Cre, this study showed that downregulation of macrophage-capping protein (MCP) reduced UFMylation of pirin (PIR), suggesting that PIR is a potential UFM1 substrate. This inhibition led to decreased transcription of GPX4 (glutathione peroxidase 4), a ferroptosis biomarker, and enhanced the cytoplasmic release of HMGB1 (high mobility group box 1). The reduced GPX4 levels initiate ferroptosis, while the released HMGB1 promotes pro-inflammatory M1-like macrophage polarization. Consequently, therapeutic targeting of MCP (monocyte chemoattractant protein 1) not only triggered ferroptosis but also activated antitumor, pro-inflammatory responses, offering a dual antitumor effect. Moreover, a nanosystem for specifically silencing MCP was developed, which provided a novel approach for PDAC treatment (109).

### PD-1 UFMylation

PD-1 and PD-L1 are key proteins involved in the immune system “checkpoint”, which manage immune responses to avoid attacking the body tissues. In the context of cancer, however, PD-1 and PD-L1 proteins are often exploited by tumor cells to evade immune destruction (110, 111). PD-1 is found on the surface of T-cells and acts as an immune checkpoint to keep the immune system in check. When PD-1 binds with its ligands, PD-L1 or PD-L2, it sends an inhibitory signal to T-cells, reducing their activity to attack the tumor cells (112). PD-L1 is mainly expressed on the surface of cancer cells. Targeting the interaction between PD-1 and PD-L1 is a promising strategy for cancer immunotherapy via using checkpoint inhibitors, such as anti-PD-1 antibodies (pembrolizumab and nivolumab) and anti-PD-L1 antibodies (atezolizumab) (113, 114). Recently, one study demonstrated that ablation of UFL1 in T cells inhibited PD-1 UFMylation to increase anti-tumor immunity (106). This study investigated the physiological role of UFMylation in T cells by examining mice with a conditional knockout (cKO) of Ufl1, focusing on tumor immunity. Ufl1 cKO mice demonstrate superior tumor control. Single-cell RNA sequencing analysis of these mice reveals an increase in tumor-infiltrating cytotoxic CD8+ T cells. Moreover, UFL1 regulated the UFMylation of PD-1, which in turn inhibited PD-1 ubiquitination and degradation (106). Additionally, AMPK-mediated phosphorylation of UFL1 at Thr536 impaired PD-1 UFMylation, leading to PD-1 degradation and enhanced CD8+ T cell activation. Consequently, the ablation of UFL1 in T cells diminishes PD-1 stability, promoting a robust anti-tumor immune response. This improved tumor immunity is particularly evident in the enhanced response of Ufl1 cKO mice to anti-CTLA-4 immunotherapy. This study not only clarify the significant role of UFMylation in T-cell function but also position UFL1 as a promising target for cancer therapy (106).

### PD-L1 UFMylation

Zhou et al. reported that dysregulation of PD-L1 by UFMylation disrupted tumor immune evasion (107). In this study, PD-L1 was identified as a target of UFMylation. Moreover, UFMylation contributes to the destabilization of PD-L1 by promoting its ubiquitination. Disrupting PD-L1 UFMylation through the depletion of UFL1 or UFM1, or by defective UFMylation of PD-L1 itself, stabilized PD-L1 in various human and murine cancer cell lines, which impair antitumor immunity both *in vitro* and *in vivo*. Additionally, a reduction in UFL1 expression was observed across multiple cancers. Moreover, lower UFL1 levels were associated negatively with anti-PD-1 therapy response in melanoma patients. Notably, a covalent inhibitor of UFSP2 that enhanced UFMylation activity was developed, potentially augmenting the efficacy of PD-1 blockade therapies. This work revealed a new regulatory mechanism of PD-L1, suggesting UFMylation as a novel therapeutic target in oncology (107).

### PLAC8 UFMylation

Placenta specific 8 (PLAC8) has been revealed to regulate cell growth in tumorigenesis (115). Mao et al. reported that PLCA8 inhibited cell apoptosis via activation of the PI3K/AKT/NF-κB pathway in breast cancer (116). PLAC8/MAPK (mitogen-activated protein kinase) axis regulated tamoxifen sensitivity in breast cancer, which was abrogated by curcumin-mediated protein stability change (117). Chen et al. reported that PLAC8 enhanced Adriamycin resistance by reduction of autophagy in breast cancer (118). PLAC8 was increased in TNBC and underwent UFM1-mediated modification, which enhances its stability and influences cellular proliferation. Importantly, PLAC8 modulated the immune response by regulating PD-L1 ubiquitination levels. Clinical data from breast cancer patients further revealed that PLAC8 expression was elevated in TNBC compared to non-TNBC and was associated positively with PD-L1 expression. These findings introduce a new PLAC8-regulated pathway in TNBC, offering new insights for clinical diagnosis and opening potential avenues for immunotherapeutic interventions in breast cancer subtype (119).

### UFMylation regulates RIG-I

RIG-I, an RNA-binding protein, initiates the antiviral innate immune response by activating downstream signaling through the adaptor protein MAVS, leading to the production of type I and III interferons (IFNs). This signaling cascade is localized at endoplasmic reticulum (ER)–mitochondrial contact sites. UFL1 as a component recruited to these contact sites following RIG-I activation. UFL1 and the UFMylation process are critical for IFN induction after RIG-I activation. Post RNA virus infection, UFL1 associates with the membrane-targeting protein 14–3-3ϵ, facilitating its recruitment to activated RIG-I, thus enhancing downstream immune signaling. Significantly, there was an increase in UFM1 conjugation of 14–3-3ϵ post RIG-I activation. Furthermore, the absence of UFMylation disrupts the interaction between 14–3-3ϵ and RIG-I, consequently impeding the linkage to MAVS (mitochondrial antiviral signaling protein) and the subsequent IFN-inducing signal transduction. These findings establish UFMylation as a crucial regulatory mechanism in RIG-I-mediated signaling and as a pivotal posttranslational modulator of IFN induction, which highlight potential targets for modulating antiviral immune responses (120).

## Inhibitors for UFMylation

### Metformin

Metformin is a widely used medication primarily for treating type 2 diabetes (121). It is recognized for its effectiveness, safety, and cost-efficiency, making it one of the most commonly prescribed drugs for managing diabetes around the world (122). Metformin works by improving the sensitivity of body tissues to insulin, thereby facilitating better cellular uptake of glucose, which lowers blood sugar levels (123). In addition, metformin has other potential health benefits and uses in prediabetes (124, 125), polycystic ovary syndrome (PCOS) (126, 127), aging (128, 129) and extending life span (130). Moreover, metformin has attracted interest for its potential in longevity via influencing fundamental aging factors (131). Metformin has been implicated to play a critical role in cancer treatment and prevention (132, 133). For example, metformin inhibited cell proliferation and glycolysis via regulation of ADAMTS12 in gastric cancer (134). Metformin reduced tumor cell stemness induced by paclitaxel via regulating FOXO3a in non-small-cell lung cancer (135). Metformin displayed antineoplastic functions via modulation of TGF-β and p38/ERK/MAPK signaling pathways in PTEN-deficient endometrial cancer (136).

Ferroptosis is a type of programmed cell death, which is distinct from other forms of cell death like apoptosis, necrosis, and autophagy (137). The process is heavily dependent on iron, which contributes to the production of ROS and lipid peroxidation (138). Targeting ferroptosis has been identified for an effective strategy for cancer treatment (139). Metformin has been reported to target apoptosis, necroptosis and ferroptosis in breast cancer cells (140). Moreover, metformin targeted miR-324-3p and GPX4, leading to induction of ferroptosis in breast cancer (141). One study showed that metformin inhibited autophagy via influencing lncRNA H19 and resulted in induction of ferroptosis in breast cancer (142). Another study showed that metformin induced ferroptosis and increased sorafenib sensitivity via regulation of ATF4 (activating transcription factor 4) and STAT3 (signal transducer and activator of transcription 3) in hepatocellular carcinoma cells (143). Metformin regulated the Nrf2/HO-1 signaling pathway and facilitated ferroptosis in lung cancer (144). Recently, Yang et al. reported that metformin mediated ferroptosis via suppression of UFMylation of SLC7A11 in breast cancer (145). Metformin promoted ferroptosis independently of AMPK to inhibit tumor growth. Metformin elevated levels of Fe^2+^ and lipid reactive oxygen species within cells. Metformin disrupted the stability of the protein SLC7A11, a key regulator of ferroptosis, by impeding its UFMylation. Additionally, when combined with sulfasalazine, metformin synergistically enhanced ferroptosis and suppressed the growth of breast cancer cells (145). Hence, metformin inhibited UFMylation of SLC7A11 in breast cancer.

### UBA5 inhibitor

One group developed a selective micromolar inhibitor of UBA5, compound 8.5, to block UFM1 protein conjugation. This group introduced a new organometallic inhibitor that features a core scaffold incorporating adenosine and zinc(II)cyclen. The inhibitor targets UBA5 selectively and noncompetitively, distinguishing itself from other E1 enzymes and a broad spectrum of human kinases. *In vitro* data showed that this inhibitor of UBA5 selectively hindered the proliferation of cancer cells, particularly effective at concentrations above 50μM in cells with elevated levels of UBA5. This inhibitor could offer potential insights into therapeutic interventions via regulation of UFM1 pathway (146). Another group used chemoproteomic screening of covalent ligands and found that UBA5 might be a potential therapy target for pancreatic cancer. Moreover, this research led to the discovery of DKM 2-93, a covalent ligand that reduced cell survival and tumor growth *in vivo* in pancreatic cancer. DKM 2-93 achieved its therapeutic effects by covalently modifying the catalytic cysteine of UBA5, thereby inhibiting its function in activating the UFM1 for protein UFMylation. UBA5 could be a potent target for pancreatic cancer therapy and DKM 2-93 might be a relatively selective inhibitor of UBA5 (74). Fang et al. reported the properties of usenamine A, a natural compound derived from the lichen Usnea longissimi, known for its inhibitory effects on UBA5. *In vitro* studies demonstrated that usenamine A effectively inhibits cell proliferation, induces G2/M phase arrest, autophagy, and endoplasmic reticulum stress in breast cancer cells. These findings suggest that usenamine A holds potential as a therapeutic agent to inhibit the breast tumorigenesis and progression in part via regulation of UBA5 (147).

## Conclusions and perspectives

**Figure 2:**
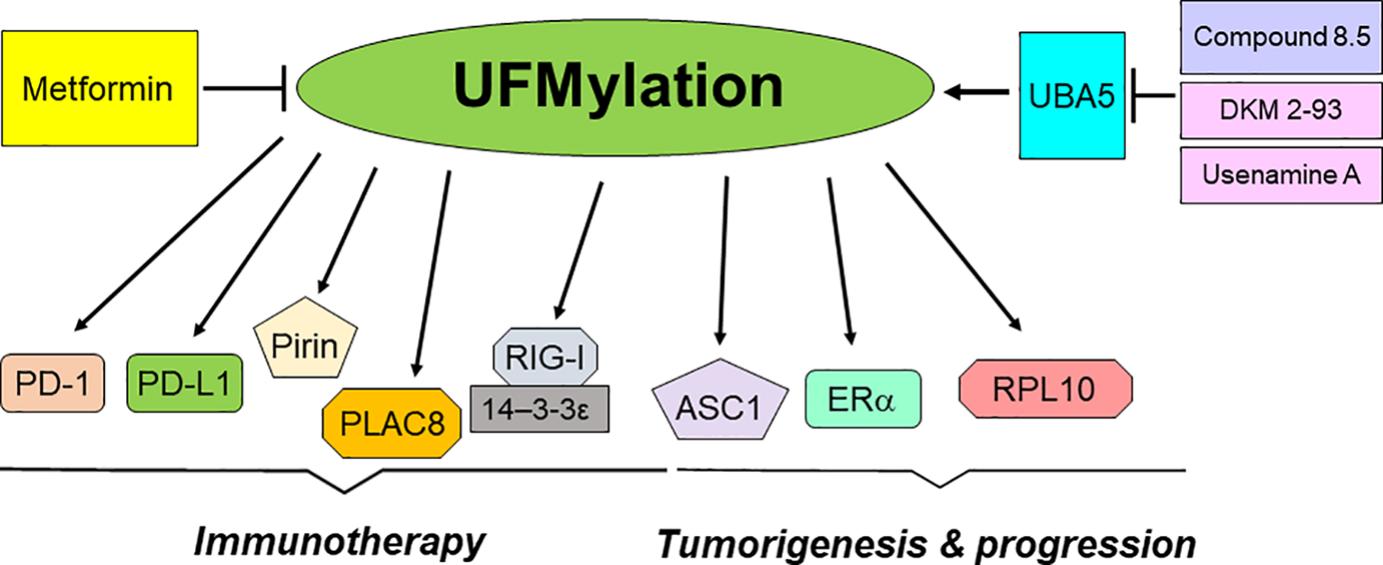
UFMylation in regulation of tumorigenesis and immunotherapy.

In conclusion, UFMylation plays an essential role in the development and progression of tumors (**Table 1**). Moreover, UFMylation is critically involved in regulation of cancer immunotherapy (**Table 2**). Utilizing UFMylation as a target may enhance the efficacy of cancer treatment and immunotherapy (**Figure 2**). As UFMylation emerges as a critical PTM with substantial implications in cellular regulation and disease, future research directions are poised to expand our understanding and harness this pathway therapeutically. UFMylation of PTIP (Pax2 transactivation domain interacting protein) was reported to confer chemoresistance in BRCA1-deficine cells (148). Further detailed studies are needed to delineate the precise molecular mechanisms by which UFMylation influences cellular processes such as protein stability, signal transduction, and stress responses. Advanced techniques such as cryo-electron microscopy and single-molecule fluorescence could provide deeper insights into the structural and functional dynamics of UFMylation at the molecular level. Although a link between UFMylation and diseases like cancer, neurodegeneration, and inflammatory disorders has been established, future research should focus on identifying specific UFMylation-related pathways that are dysregulated in these conditions. This involves comprehensive proteomic studies and genetic screening to map out the UFMylation substrates and their pathophysiological roles in various diseases. In addition, there is a compelling need to develop specific inhibitors or enhancers of UFMylation enzymes as potential therapeutic agents. Drug discovery efforts should be intensified to identify small molecules or biologics that can modulate the UFMylation pathway with high specificity and low toxicity. Given its role in critical cellular functions, UFMylation could serve as a biomarker for disease progression or therapeutic response, particularly in cancer and immune-related conditions. Research should be directed towards validating UFMylation-related proteins or modifications as diagnostic, prognostic, or predictive biomarkers. Combining UFMylation studies with other omics approaches (genomics, transcriptomics, metabolomics) can yield comprehensive insights into how UFMylation interacts with other cellular pathways and influences complex biological systems. Such integrative studies would be crucial for constructing a holistic model of cellular regulation and disease manifestation. Finally, the translation of bench research to bedside application will require rigorous clinical trials to evaluate the safety and efficacy of UFMylation-targeted therapies. In summary, research on UFMylation can advance from basic science to clinical applications, offering new strategies for treating a variety of diseases and enhancing our understanding of cellular homeostasis.

**Table 1:** Role of UFMylation in tumorigenesis.

Item	Mechanisms	Functions	Refs
Breast cancer	ASC1 UFMylation leads to activation of ERα in response to 17β-estradiol.	Promotes tumor development.	(42)
Breast cancer	ERα UFMylation enhances ERα’s stability and transactivation capabilities.	Promotes breast cancer progression.	(43)
Glioblastoma	UBA5, UFC1, UFL1, and UFSP2 knockouts reduced cell fitness. Depletion of UFC1 and UFSP2 promoted CHOP expression.	GBM stem cell maintenance and cell proliferation.	(54)
Renal cancer	Upregulation of active UFM1 partially counteracts the anti-tumor effects of sunitinib.	UFMylation might serve as target.	(61)
Colon cancer	By knocking down UFSP2, there was an increase in the expression of PCNA, MCM2, CDK4, CCND1, RPL26.	Knockdown of UFSP2 enhances the growth rates of colon cancer cells.	(67)
Pancreatic cancer	RPL10 UFMylation enhances cell proliferation and promoted cancer cell stemness via upregulation of KLF4.	UFMylation of RPL10 promotes the stemness, contributing to the progression.	(75)
OSCC	Reducing UFM1 expression suppressed the cell proliferation, attenuated cell migration, and invasion.	UFM1 was linked to Th17 cells, T helper cells, and cytotoxic cells.	(82)
HCC	Loss of Ufl1 or Ufbp1 leads to enhanced mTOR pathway and HCC development.	Ufl1 and Ufbp1 prevent liver fibrosis, steatohepatitis, and HCC by inhibition of mTOR.	(89)
Gastric cancer	UFM1 enhances the PDK1 ubiquitination level, leading to reduction of the PDK1 protein level, thereby reducing the AKT phosphorylation at Ser473.	Upregulation of UFM1 inhibits migration and invasion abilities *In vivo* study further identified the tumor suppressive function of UFM1.	(96)
Gastric cancer	UFBP1 decreases AKR1Cs expression and Nrf2 transcriptional activity, promoting Nrf2 degradation.	UFBP1 enhances the sensitivity of gastric cancer cells to cisplatin through the Nrf2/AKR1C axis.	(97)

**Table 2:** Role of UFMylation in immunotherapy.

Item	Mechanism	Functions	Ref
Pirin	Downregulation of MCP reduces UFMylation of pirin. This inhibition leads to decreased transcription of GPX4, and enhances the cytoplasmic release of HMGB1.	The reduced GPX4 levels initiate ferroptosis, and release HMGB1 promotes pro-inflammatory M1-like macrophage polarization.	(109)
PD-1	UFL1 regulates PD-1 UFMylation, which inhibits PD-1 degradation. AMPK-mediated UFL1 phosphorylation impairs PD-1 UFMylation and PD-1 degradation.	Ablation of UFL1 in T cells inhibited PD-1 UFMylation to increase anti-tumor immunity.	(106)
PD-L1	PD-L1 UFMylation contributes to the destabilization of PD-L1 by promoting its ubiquitination.	Disrupting PD-L1 UFMylation stabilizes PD-L1 and impairs antitumor immunity.	(107)
PLAC8	PLAC8 modulated the immune response by regulating PD-L1 ubiquitination levels.	PLAC8 expression is elevated in TNBC and is associated positively with PD-L1 expression.	(119)
RIG-I	UFL1 associates with the 14–3-3ϵ, facilitating its recruitment to activated RIG-I, thus enhancing downstream immune signaling.	Absence of UFMylation disrupts the interaction between 14–3-3ϵ and RIG-I, impeding the linkage to MAVS and IFN-inducing signal transduction.	(120)
